# Aggregation‐Induced Absorption Enhancement for Deep Near‐Infrared II Photoacoustic Imaging of Brain Gliomas In Vivo

**DOI:** 10.1002/advs.201801615

**Published:** 2019-01-16

**Authors:** Yajing Liu, Huanhuan Liu, Huixiang Yan, Yingchao Liu, Jinsen Zhang, Wenjun Shan, Puxiang Lai, Honghui Li, Lei Ren, Zijing Li, Liming Nie

**Affiliations:** ^1^ State Key Laboratory of Molecular Vaccinology and Molecular Diagnostics & Center for Molecular Imaging and Translational Medicine School of Public Health Xiamen University Xiamen 361102 China; ^2^ Department of Ultrasonography Second Clinical College of Jinan University Shenzhen People's Hospital Shenzhen 518020 China; ^3^ Department of Neurosurgery Provincial Hospital Affiliated to Shandong University Shandong 250021 China; ^4^ Department of Neurosurgery Huashan Hospital Fudan University Shanghai 200040 China; ^5^ Department of Biomaterials College of Materials Xiamen University Xiamen 361005 China; ^6^ Department of Biomedical Engineering The Hong Kong Polytechnic University Hong Kong 999077 China

**Keywords:** aggregation‐induced absorption enhancement, brain gliomas, deep photoacoustic imaging, mesoionic dyes, second near‐infrared window

## Abstract

The delineation of brain gliomas margins still poses challenges to precise imaging and targeted therapy, mainly due to strong light attenuation of the skull and high background interference. With deep penetration and high sensitivity, photoacoustic (PA) imaging (PAI) in the second near‐infrared (NIR II) window holds great potential for brain gliomas imaging. Herein, mesoionic dye A1094 encapsulated in Arg‐Gly‐Asp‐modified hepatitis B virus core protein (RGD‐HBc) is designed and synthesized for effective NIR II PAI of brain gliomas. An aggregation‐induced absorption enhancement mechanism is discovered in vitro and in vivo. It is also demonstrated that A1094@RGD‐HBc, with an enhanced absorption in the NIR II window, displays ninefold PA signal amplification in vivo, allowing for precise PAI of the brain gliomas at a depth up to 5.9 mm. In addition, with the application of abovementioned agent, high‐resolution PAI and ultrasensitive single photon emission computed tomography images of brain gliomas are acquired with accurate co‐localization. Collectively, the results suggest great promise of A1094@RGD‐HBc for diagnostic imaging and precise delineation of brain gliomas in clinical applications.

Brain gliomas are the most lethal malignant intracranial neoplasms worldwide, associated with poor prognosis and high mortality rates.^1^ Currently, aggressive surgical resection after imaging detection is the standard treatment in the clinic.^2^ However, current diagnosis and treatment is limited by inaccurate assessment of tumor locations and ambiguous surgical margins in the brain.^3^ Moreover, strong light attenuation and scattering in the skull and parenchyma tend to impede light penetration, making it difficult to employ free‐space optical methods to probe deep brain regions.^4^ Therefore, a highly sensitive imaging method that can accurately delineate the surgical margins of deep brain gliomas is urgently demanded.

Photoacoustic (PA) imaging (PAI), with rich contrasts and high spatial resolution in a single modality, has gained immense attention of scientists and physicians in recent years.^5^ This novel technique with effective PA agents is useful for disease surveillance, particularly for early stage theranostics and noninvasive tumor imaging.^6^ As previously reported, copper sulfide was developed as a PAI agent for in vivo mice brain imaging at 1064 nm.^7^ However, high doses of the agent were necessitated to enhance imaging contrasts, which may induce severe adverse effects or toxicities, since gray matter exhibits higher scattering and stronger absorption than the surrounding tissues (including muscle and adipose tissue).^8^ Another challenge of brain gliomas detection and treatment is how to deliver agents to the tumor tissues. The blood–brain barrier (BBB) is a main obstacle to drug delivery, especially for the majority of inorganic absorbing agents with low biodegradability and compositions complexity.^9^ Thus, to overcome these challenges, the development of a safe and effective glioma‐targeting organic probe with strong absorption in the second near‐infrared window (NIR II) is exceptionally critical for deep PAI of brain diseases.^10^

Herein, a group of π–π‐conjugated mesoionic organic molecules (including A1094), which exhibited excellent aggregation‐induced absorption enhancement (AIAE) performance when aggregating in biocompatible carriers, was discovered for deep PAI (**Scheme** **1**a). In contrast to aggregation‐induced emission phenomenon (AIE),^11^ AIAE refers to a photophysical phenomenon with obvious absorption efficiency enhancement other than fluorescence enhancement when the monomers form aggregates. The mechanism is attributed to the energy gap resonance enhancement between highest occupied molecular orbital (HOMO) and lowest unoccupied molecular orbital (LUMO) (Scheme 1b). The AIAE of chromophores caused by aggregation enables energy transfer intermolecularly instead of photon emission.^12^ AIAE occurs when dye molecules gather together for longer distance energy gap coupling at a lower concentration instead of shorter distance energy gap coupling at a higher concentration in J/H‐aggregates.^13^ In brief, energy transfer of delocalized compounds results in increased optical absorption through AIAE.

**Scheme 1:**
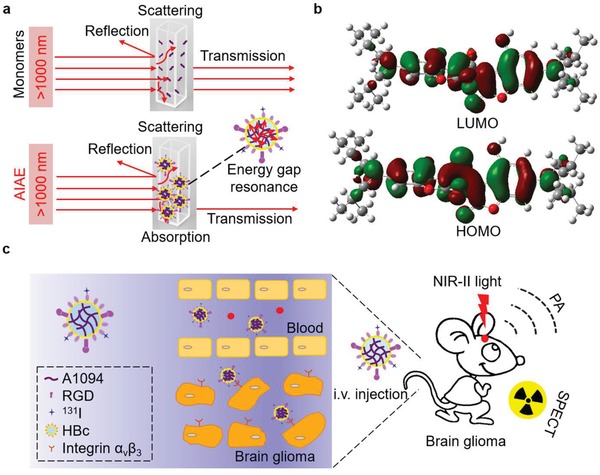
AIAE mechanism and application of A1094 aggregates for brain gliomas imaging. a) Schematic representation of absorber‐loaded probe with AIAE performance. b) The density functional theory calculation for the HOMO and LUMO of A1094 using B3LYP functional and 6–311G (d, p) basis set with the solvent model density model. c) Application for accurate mapping of orthotopic brain gliomas.

In our study, the AIAE phenomenon was confirmed through ingenious design and molecular engineering. At a proper concentration range, nonlinear absorption in the NIR II window was revealed in A1094@Arg‐Gly‐Asp‐hepatitis B virus core (A1094@RGD‐HBc) to allow deep PAI. The AIAE phenomenon was applied for the first time to promote highly efficient PAI at a very low dose. Deep NIR II PAI and single photon emission computed tomography (SPECT) of orthotopic brain gliomas were realized with excellent correspondence (Scheme 1c).

The synthetic processes of the organic molecule A1094 were illustrated in Scheme S1 in the Supporting Information. The physicochemical properties of intermediates and A1094 dye were shown in Figures S1–S8 in the Supporting Information. The HOMO and LUMO orbital energies of A1094 were found to be −4.47 and −3.57 eV, respectively, indicating a small energy band gap for the NIR II absorption (Figure S9, Supporting Information). A1094 displayed a strong absorption peak at 1094 nm in **Figure** **1**a and was well dissolved in organic solvents including ethanol, methanol, and dimethyl sulfoxide (DMSO), with the molar extinction coefficients of 5.2E3, 2.3E4, and 1.4E4, respectively (Figure S10, Supporting Information). A1094 exhibited different absorption levels at different pHs. The superior absorption in acidic pHs (4.1–5.9) implied the suitability of A1094 as a probe for tumor acidic microenvironment imaging (Figure S11, Supporting Information). Furthermore, A1094 was proved to be stable in dichloromethane under white light or laser irradiation (Figures S12 and S13, Supporting Information). After 5 days' storage, it was well dispersed in DMSO (Figure S14a, Supporting Information). Encouraged by the intense NIR II absorption, its PA performance was evaluated in DMSO (Figure 1b). The PA property of A1094 exhibited high detection sensitivity, even at a concentration as low as 62.5 µg mL^−1^ (Figure 1c).

**Figure 1:**
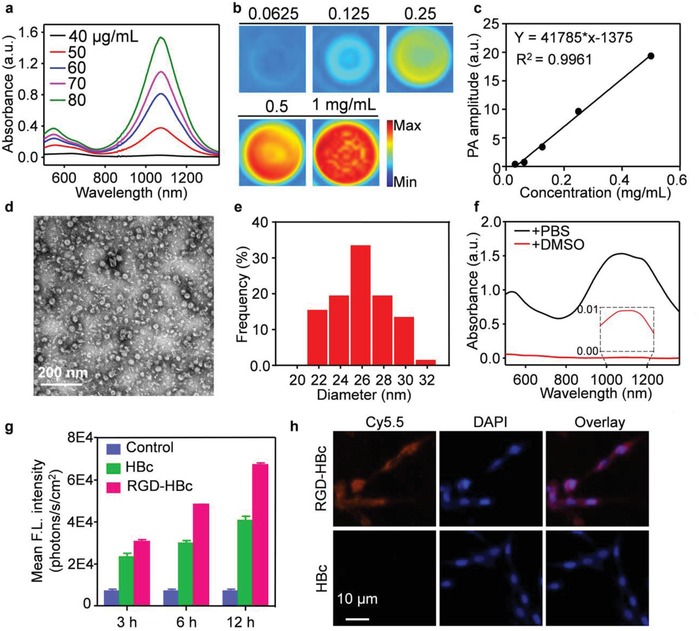
Characterization of A1094@RGD‐HBc. a) Absorption spectra of A1094 in methanol at graded concentrations (40–80 µg mL^−1^). b) PA images of A1094 at different concentrations (0.0625, 0.125, 0.25, 0.5, 1.0 mg mL^−1^) at 950 nm in DMSO. c) PA amplitudes as a function of A1094 concentrations in DMSO. d) Transmission electron microscope of A1094@RGD‐HBc. e) Dynamic light scattering of A1094@RGD‐HBc. f) Absorption spectra of A1094@RGD‐HBc before and after 90% DMSO destroying the protein. g) Flow cytometry analyses of U87MG cell after incubation with RGD‐HBc and HBc at different time points. h) Fluorescence images of U87MG cell after incubation with Cy5.5 labeled A1094@RGD‐HBc and A1094@HBc.

To construct efficient agents for biomedical applications, A1094 was encapsulated into RGD‐HBc protein. Active expression of RGD‐HBc was confirmed with sodium dodecyl sulfate (SDS) polyacrylamide gel electrophoresis (PAGE) and western blot analyses (Figure S15, Supporting Information). Both dynamic light scattering and transmission electron microscopy illustrated that the diameter of A1094@RGD‐HBc was around 30 nm (Figure 1d,e). In addition, the high stability of the synthesized agent was demonstrated before and after white light irradiation in phosphate‐buffered saline (PBS; Figures S14b and S16, Supporting Information). Under 980 nm laser irradiation (0.5 W cm^−2^, 10 min), the excellent photothermal stability of A1094@RGD‐HBc was illustrated in Figure S17 in the Supporting Information.

After loading A1094 into the RGD‐HBc protein, AIAE in the NIR II region was remarkably noticed (Figure 1f). A strong absorption intensity of A1094@RGD‐HBc was observed even at a very low concentration (25 µg mL^−1^) in a nonlinear relationship (Figure S18a,b, Supporting Information). To further illustrate the mechanism of AIAE, A1094@RGD‐HBc was then dissociated with DMSO, making the release of A1094 from RGD‐HBc protein, which resulted in a consequent reduction in the NIR II absorption. Meanwhile, no AIAE phenomenon was found in Oil Red O@RGD‐HBc as shown in Figure S19 in the Supporting Information.

In order to study the selectivity of RGD toward the human primary glioblastoma cell line (U87MG), U87MG cells were incubated with RGD‐HBc, HBc, and PBS, respectively. After incubation with RGD‐HBc for 12 h, the fluorescence intensity of the treated cells was found to be 9.6 times brighter than that of PBS‐treated cells, and 1.6 times brighter than that of HBc‐treated cells (Figure 1g). Confocal microscopy results illustrated more specific cellular uptake of A1094@RGD‐HBc than A1094@HBc (Figure 1h). Furthermore, more than 80% cells survived after 12 h incubation with A1094@RGD‐HBc at various concentrations (0–250 µg mL^−1^), confirming the low cytotoxicity of the probe (Figure S20, Supporting Information).

Considering the challenge of BBB, the biodistribution of A1094@RGD‐HBc in U87MG/Luc orthotopic model was further evaluated by NIR fluorescence imaging (Figure S21, Supporting Information). Strong fluorescence signal was observed in the brain of tumor‐bearing mice at 2 h post‐injection, while no obvious NIR fluorescence signal could be detected in the sham and normal groups (Figure S21a, Supporting Information). These results further displayed a better brain tumor‐targeting efficacy of A1094@RGD‐HBc in tumor‐bearing mice than in the normal group (Figure S21b, Supporting Information). Subsequently, magnetic resonance imaging and hematoxylin‐eosin (H&E) staining were implemented to further illustrate the location and histology of the tumors (Figure S22, Supporting Information).

The efficient targeting property and highly enhanced absorption in the NIR II region of A1094@RGD‐HBc motivated us to further explore its PA performance at different wavelengths (800–1400 nm; **Figure** **2**a). After laser energy correction, the results revealed that the PA amplitudes of aggregates at 1200–1300 nm were significantly stronger than those at 800–950 nm (Figure 2c and Figure S23, Supporting Information). Then, these aggregates were disposed to elucidate AIAE performance. Figure 2d shows significant PA amplitudes at 1200 nm with AIAE property. As anticipated, weak PA signals exhibited at the depolymerized state, suggesting that the enhanced PA amplitudes were induced by A1094 aggregation. Next, A1094@RGD‐HBc was subcutaneously injected into mice skin. Superior PA signals under excitation around 1200 nm were detected as expected (Figure 2b). Conversely, the contrast without AIAE property exhibited low PA amplitudes in vivo. The aggregation of A1094 in RGD‐HBc could induce ninefold absorption enhancement in vivo, implying its advantage as an ideal agent candidate for PAI of deep brain tumors (Figure 2e). Taking the light attenuation into account, which has been proved to be relatively weak at 1064 nm in the mouse skull,^14^ 1064 nm was selected for the following PAI of the orthotopic brain gliomas in vivo.

**Figure 2:**
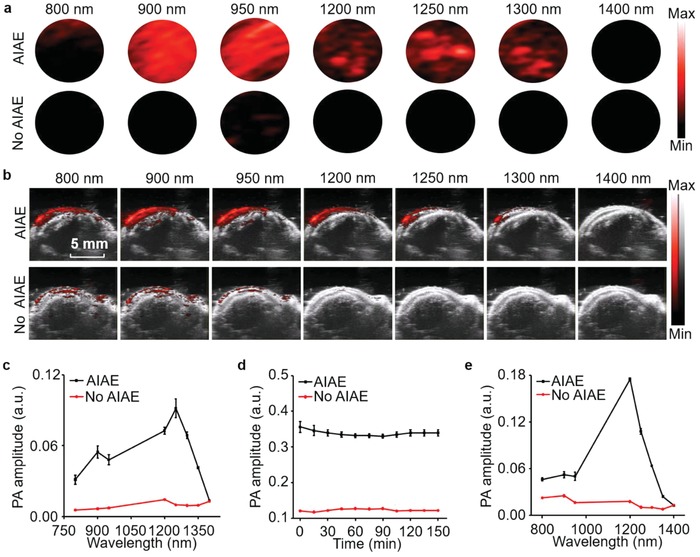
AIAE performance of A1094@RGD‐HBc. PAI of the probe before and after depolymerization at 800–1400 nm a) in vitro and b) in vivo. c) Corresponding PA amplitudes of (a). d) PA signals stability of A1094@RGD‐HBc at 1200 nm in 150 min. e) Corresponding PA amplitudes of (b).

Then, the highly efficient contrast agent with enhanced PA performance was evaluated in brain tumor‐bearing mice by a home‐made acoustic‐resolution photoacoustic microscopy (AR‐PAM; **Figure** **3**a). A low dose of A1094@RGD‐HBc (100 µg mL^−1^) was administrated by intravenous injection. PA model was applied to monitor the accumulation of A1094@RGD‐HBc, while ultrasound (US) model was applied to reveal tumor locations (Figure 3b–d). Notably, the B‐scans of PA and US showed that the depth of the tumor from brain skin was 5.9 mm (Figure 3e–g), which was 1.47 times deeper than the result of a previously reported study.^14^ It is worth noting that owing to the AIAE property, the dose of A1094@RGD‐HBc was only a quarter of ICG@RGD‐HBc when employed for PAI.^15^

**Figure 3:**
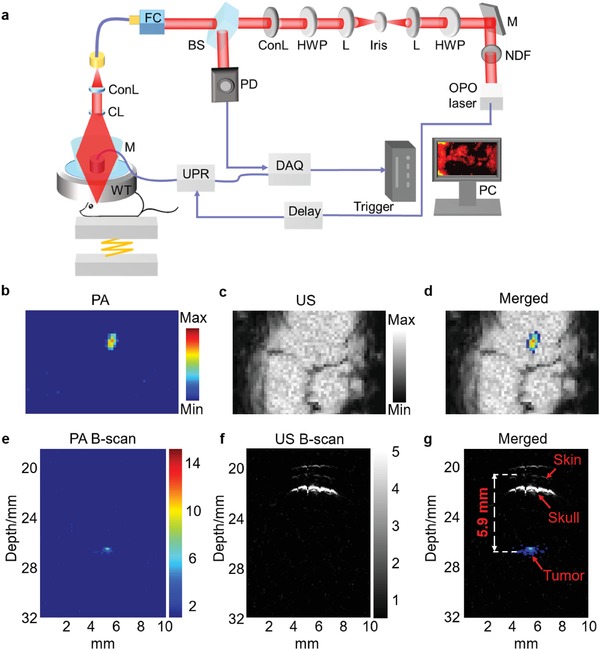
Noninvasive PAI of glioma‐bearing mice. a) AR‐PAM system for imaging of U87MG tumor‐bearing mice. OPO: optical parametric oscillator; NDF: neutral density filter; M: mirror; HWP: half‐wave plate; L: lens; ConL: convex lens; BS: beam splitter; FC: fiber coupler; PD: photodiode; CL: conical lens; WT: water tank; UPR: ultrasonic pulser/receiver; DAQ: data acquisition card; PC: personal computer. b) PA, c) US, and d) merged images of the brain in U87 tumor‐bearing mice. The corresponding B‐scans of e) PA, f) US, and g) merged images of tumors 2 h post‐injection.

To further verify the enhanced PA signals in tumors, maximum amplitude projection images of mice brain were obtained 2 h after injection (**Figure** **4**a). The pseudocolor revealed enhanced PA signals of the tumor obtained at 1064 nm while the gray depicted the co‐registered US image of the skull. The results illustrated the excellent performance of A1094@RGD‐HBc as an efficient PA agent for brain tumors. Labeled with ^131^I, the synthesized ^131^I‐A1094@RGD‐HBc was used for ultrasensitive SPECT imaging of gliomas (Figure S24, Supporting Information). The fused microSPECT/CT image revealed enhanced radioactive uptake in tumors at 2 h post‐injection (Figure 4b), which was consistent with the PAI result. After imaging, brain tissues were used for autoradiography study and major organs were collected for H&E staining. Compared with normal brain slices, the tumor brain slices showed higher radioactive uptake, reflecting an excellent specificity (Figure 4c). Moreover, no apparent cellular changes were observed, indicating good biocompatibility of the probe in vivo (Figure S25, Supporting Information). These results suggested that ^131^I‐A1094@RGD‐HBc, with extraordinary efficiency, specificity, and biocompatibility, could be developed as an ideal contrast agent for deep PAI and ultrasensitive SPECT imaging of orthotopic brain gliomas.

**Figure 4:**
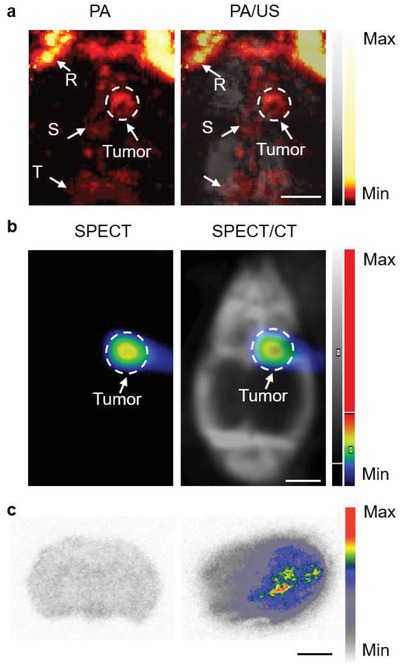
In vivo PA/US and microSPECT/CT imaging. a) PA/US images and b) microSPECT/CT images of the brain of U87MG tumor‐bearing mice at 2 h after injection of ^131^I radiolabeled A1094@RGD‐HBc. c) The autoradiography images of the control group (left) and tumor‐bearing group (right) after injection of ^131^I‐A1094@RGD‐HBc. Scale bar = 2 mm. R: rostral rhinal vein; S: sagittal sinus; T: transverse sinus.

In summary, the present study for the first time demonstrated a novel AIAE phenomenon and exploited it to PAI of deep brain tumors in the NIR II window. In the conventional chromophores, aggregation usually leads to adverse effects on their imaging applications because of quenching effect. Nevertheless, the AIAE absorbers avoid this problem and take advantage of the less concentrated aggregation. After encapsulation into protein carriers within a restricted space, the A1094@RGD‐HBc exhibited enhanced NIR II absorption and powerful PA contrast enhancement capability, making it promising for applications in the deep orthotopic brain tumor imaging. The AIAE agent also exhibited excellent photostability, specific tumor targeting, and highly efficient concentration‐independent absorption, which exceeded many inorganic absorbers, such as gold nanostars.[[qv: 6b]] The AIAE effect provides an excellent opportunity for researchers to harness this effect of light‐absorbing processes for suitable absorber aggregates. In the future, our study will focus on developing a series of novel and highly efficient AIAE contrast agents as well as extending their advantage of deep PAI for clinical translation.

## Conflict of Interest

The authors declare no conflict of interest.

## Supporting information

Supplementary
