# Supplementary material for: Aggregation‐Induced Absorption Enhancement for Deep Near‐Infrared II Photoacoustic Imaging of Brain Gliomas In Vivo
Source: Adv Sci (Weinh). 2019 Jan 16;6(8):1801615. doi: 10.1002/advs.201801615 (PMC6469237; doi:10.1002/advs.201801615)
Supplement: Supplementary file 1 — Supplementary [file ADVS-6-1801615-s001.pdf]

Copyright WILEY-VCH Verlag GmbH & Co. KGaA, 69469 Weinheim, Germany, 2019.

ADVANCED SCIENCE
Open Access

# Supporting Information

for *Adv. Sci.,* DOI: 10.1002/advs.201801615

# Aggregation-Induced Absorption Enhancement for Deep Near-Infrared II Photoacoustic Imaging of Brain Gliomas In Vivo

*Yajing Liu, Huanhuan Liu, Huixiang Yan, Yingchao Liu, Jinsen Zhang, Wenjun Shan, Puxiang Lai, Honghui Li, Lei Ren, Zijing Li,* and Liming Nie**

Copyright WILEY-VCH Verlag GmbH & Co. KGaA, 69469 Weinheim, Germany, 2019.

# Supporting Information

## Aggregation induced absorption enhancement for deep near-infrared II photoacoustic imaging of brain gliomas *in vivo*

*Yajing Liu[+], Huanhuan Liu[+], Huixiang Yan, Yingchao Liu, Jinsen Zhang, Wenjun Shan, Honghui Li, Puxiang Lai, Lei Ren, Zijing Li,* and Liming Nie**

## Table of Contents

**Materials and Methods**

**Scheme S1**. Synthetic route of A1094.

**Figure S1.** Mass spectrum of compound **1**.

**Figure S2.** $^{1}$H NMR spectrum of compound **1**.

**Figure S3.** $^{13}$C NMR spectrum of compound **1**.

**Figure S4.** Mass spectrum of compound **2** (A1094).

**Figure S5.** $^{1}$H NMR spectrum of compound **2** (A1094).

**Figure S6.** $^{13}$C NMR spectrum of compound **2** (A1094).

**Figure S7.** FTIR spectroscopy of compound **2** (A1094).

**Figure S8.** FTIR spectroscopy of compound **2** (A1094) in the scope of 500 to 9500 cm$^{-1}$.

**Figure S9.** The density functional theory (DFT) calculation for HOMO and LUMO of A1094.

**Figure S10.** Absorption spectra of A1094 in different solutions.

**Figure S11.** The pH stability of A1094.

**Figure S12.** Light stability of A1094.

**Figure S13.** Photothermal stability of A1094.

**Figure S14.** Apparent stabilites in solutions.

**Figure S15.** Expression of RGD-HBc-NS5A protein.

**Figure S16.** Light stability of A1094@RGD-HBc.

**Figure S17.** Photothermal stability of A1094@RGD-HBc

**Figure S18.** Absorption of A1094@RGD-HBc.

**Figure S19.** Light absorption of Oil Red O@ RGD-HBc.

**Figure S20.** Cell viability.

**Figure S21.** Biodistribution of A1094@RGD-HBc in glioma-bearing mice.

**Figure S22.** Diagnosis of brain cancer.

**Figure S23.** The energy curves of photoacoustic at different wavelengths.

**Figure S24.** The labeling rate of $^{131}$I-A1094@RGD-HBc nanoparticles.

**Figure S25.** H&E stain showing major organs after 7 days of A1094@RGD-HBc administration (magnification: 200×).

## Materials and Methods

All the commercially available reagents were purchased from J&K Scientific Ltd. (Shanghai, China) and used without further purification. The $^{1}$H NMR and $^{13}$C NMR spectra were obtained on an Avance III spectrometer (600 MHz, Bruker, USA) or a WNMR-I spectrometer (400 MHz, Zhongke-Niujin, China). Mass spectra (MS) and high-resolution mass spectra (HRMS) were acquired from a Xevo G2-XS Tof (Waters, USA) equipped with electrospray ionization (ESI) or a Q Exactive LC-MS/MS instrument (Thermo Fisher, USA). The nanoparticle size was measured using dynamic light scattering (Zetasizer Nano ZS90, U.K.) and transmission electron microscopy (Tecnai G2 Spirit, China). UV-vis-NIR spectra were obtained using a Lambda 950 UV-vis-NIR spectrophotometer (PerkinElmer, USA).

The brain tumor volume was evaluated using a 9.4T BioSpec MRI scanner (Bruker, USA). PAI was performed with a home-made acoustic-resolution PA microscopy (AR-PAM) system (ultrasound 50 MHz, laser repetition rate 5 kHz, China) and commercial Visualsonics LAZR-X Vevo (ultrasound 55 MHz, laser repetition rate 20 Hz, Fujifilm, Japan). MicroSPECT/CT imaging was conducted using a nanoScan scanner (Mediso Medical Imaging System, Hungary) equipped with pinhole collimator under standard animal scan procedure.

**Synthesis of 5-[bis(2-methylpropyl) amino]-1,3-benzenediol (1).** Compound **1** was obtained following a modified version of the previously reported protocol as a brown-gray oily liquid solid (2.18 g, 92% yield). HRMS (ESI) m/z：[M + H]$^{+}$ calculated for $C_{14}H_{23}NO_2$, 238.17; found 238.11. $^{1}$H NMR (400 MHz, DMSO-$D_6$, δ): 8.75 (s, 2H, OH), 5.57 (s, 2H, Ar H), 5.52 (s, 1H, Ar H), 3.04 (d, *J* = 7.2Hz, 4H, $CH_2$), 2.00 (m, 2H, CH), 0.86 (d, *J* = 6.6 Hz, 13H, $CH_3$). $^{13}$C NMR (100 MHz, $CDCl_3$) δ: 159 (Ar C-OH), 150 (Ar C-N), 91.7 (Ar C), 91.2 (Ar C), 60 ($CH_2$), 26 (CH), 20 ($CH_3$).

**Synthesis of (*E*)-(6-(diisobutylamino)-4-hydroxy-2-oxobenzofuran-3(2*H*)-ylidene)(6-(diisobutyliminio)-4-hydroxy-2-oxo-2,6-dihydrobenzofuran-3-yl)methanolate (2, A1094).** Compound A1094 was obtained following a modified version of the previously reported protocol. Under an argon atmosphere, 5-[bis(2-methylpropyl) amino]-1,3-benzenediol (1.30 g, 5.48 mmol) and croconic acid (0.389 g, 2.73 mmol) were dissolved in dry DMSO (36 mL), stirred at room temperature for 1 h, then were refluxed for 32 min. The brown-purple solution was rapidly cooled with liquid nitrogen, and the product was purified by a flash precipitation method to give a black purple solid (0.124 g, 7.8%). HRMS (ESI) m/z： $[M - H]^-$ calculated for $C_{33}H_{42}N_2O_7$, 577.30; found 577.34. $^1H$ NMR (600 MHz, $CDCl_3$, δ): 13.82 (s, 2H, OH), 6.06 (s, 2H, Ar H), 5.88 (s, 2H, Ar H), 3.35 (s, 8H, $NCH_2$), 2.15 (m, 4H, CH), 0.96 (d, $J$ = 6.6 Hz, 24H, $CH_3$). $^{13}C$ NMR (150 MHz, $CDCl_3$) δ: 159.7 (s, C=O), 157.0 (s, $C_{arom}$-O), 156.9 (s, $C_{arom}$-N), 95.9 (s, 2C, $C_{arom}$), 90.6 (s, 4C, $C_{arom}$), 61.2 (s, $NCH_2$), 31.4 (s, CH), 29.7 (s, CH), 28.47 (s, CH), 20 (s, $CH_3$).

**Cellular uptake.** U87MG cells ($1\times10^4$ cells/well) were plated into 6-well dishes and cultured at 37 ℃ for 24 h. After incubation with Cy5.5-A1094@HBc and Cy5.5-A1094@RGD-HBc for different times (0, 3, 6, 12 h), the cells were washed with PBS. For fluorescent detection, the harvested cells were stained with DAPI. Fluorescent images were acquired with a confocal microscope. For flow cytometry analysis, cells suspension was detected by a flow cytometry (BD Accuri™, USA).

**Preparation and characterization of A1094@RGD-HBc.** HBc VLPs were expressed and purified based on previous protocols.[1] The plasmid of HBc-NS5A or RGD-HBc-NS5A was transformed into BL21 (DE3, *E. coli* strain) and then induced by isopropyl-β-d-thiogalactoside. After centrifugation and lysis, expression products were purified by chromatography (DEAE chromatography, and Sepharose CL 4B chromatography). The expressed fusion protein was then analyzed by western blotting. A1094@RGD-HBc VLPs were prepared as follows: the genetically modified VLPs were

depolymerized with urea buffer (6 M urea, 150 mM NaCl, 50 mM Tris-HCl, pH 8, 1% glycine) at 4 ℃ for 0.5 h. After depolymerization, A1094 solutions at graded concentrations were dissolved in acid DMSO, then mixed with dissociated proteins for 0.5 h at 4 ℃. Mixtures were dialyzed into assembly buffer (150 mM NaCl, 10 mM Tris-HCl pH 8, 1% glycine, 10% glycerol) overnight at 4 ℃ to reassemble HBc VLPs. Dialysis buffer (150 mM NaCl, 10 mM Tris-HCl, pH 8, 1% glycine) was changed for 24 h.

**Photothermal stability.** To test the photothermal effect, A1094 and A1094@RGD-HBc were exposed to 980 nm laser (0.5 W $cm^{-2}$, 10 min). During laser irradiation, the temperature was continuously monitored with a CCD camera. To study photothermal stability, the absorption spectra of A1094 and A1094@RGD-HBc were evaluated before and after being treated by laser irradiation.

**Cytotoxicity assessment of A1094@RGD-HBc.** U87MG cells ($5\times10^3$ cells/well) were seeded into a 96-well plate and cultured for 24 h. A1094@RGD-HBc was then incubated with U87MG cells at different concentrations (0, 30, 60, 130, and 250 μg/mL). After 12 h, the cytotoxicity of A1094@RGD-HBc was evaluated by CCK-8.

**Animal models.** All animal studies were performed under the animal use and care regulations approved by Center of Animal Care and Use Committee, Xiamen University. Balb/c nude mice (18-22 g) were purchased from Beijing Vital River Laboratory Animal Technology (China). Orthotopic tumor-bearing animal models were established by injecting a suspension of $2 \times 10^5$ U87MG cells in PBS (10 μL) into brain of each mouse (bregma + 1.0 mm, right lateral 2.5 mm, and depth 3.0 mm).

**Biodistribution.** The nude mice were divided into three groups for biodistribution study: (1) U87MG/Luc tumor-bearing mice, (2) the sham group, (3) healthy nude mice. The distribution of Cy5.5-

A1094@RGD-HBc was assessed by fluorescent imaging at 0, 2, 12, 24, and 36 h after intravenous injection. All animals were anesthetized with isoflurane and then injected with Cy5.5-A1094@RGD-HBc (100 μL, 100 μg/mL). The brain and whole body NIR fluorescence images were acquired with an IVIS Lumina II fluorescence imaging system (Ex/Em = 675/694 nm).

**PAI of A1094@RGD-HBc.** To explore the PA performance of A1094@RGD-HBc under different wavelengths, PA imaging was performed with a commercial Visualsonics LAZR-X Vevo system, which covers wavelengths from 680 to 970 nm and 1200 to 2000 nm.

**PAI of orthotopic tumors.** To study the PA performance of A1094@RGD-HBc in orthotopic tumors, the PA and US images of the brain tumors area were analyzed with an AR-PAM system. Before PAI experiment, the mice were anesthetized and then placed on an animal stage in prone position. Ultrasonic couplant was painted on brain for acoustic coupling. After intravenous injection of A1094@RGD-HBc, PAI was performed over 2 h.

**$^{131}$I labeling of A1094@RGD-HBc agent.** 100 μg of 1,3,4,6-tetrachloro-3a,6a-diphenylglycoluril was filmed in a 1.5 mL reaction tube. A1094@RGD-HBc protein (100 μL, 3mg/mL) was added into that reaction vessel followed by 100 μL phosphate buffer (PB). Na$^{131}$I solution (11-14 MBq, 5 μL) was added to the vessel and the reaction mixture was kept at 36 ℃ for 10 min. Labeling efficiency was detected by radioactive thin layer chromatography (TLC) analysis. $^{131}$I-A1094@RGD-HBc was formulated before injection.

**MicroSPECT imaging and autoradiography.** $^{131}$I-A1094@RGD-HBc (5.55 MBq, 100 μL) was intravenously injected to Balb/c nude mice bearing U87MG tumor. MicroSPECT/CT imaging of both

the brain and the body were performed at 2, 4, 6 and 12 h post injection, respectively. All the images were reconstructed and processed using Tera-TomoTM and VivoQuantTM software, respectively.

For autoradiography, the brain of normal mice and tumor-bearing mice were taken 2 h post injection. Brain sections were cut coronally at -20 ℃ by a cryostat microtome (SLEE MEV, Labsun, China). 20-μm-thick slices were mounted on glass slides. Autoradiographs were acquired by exposing these slices to phosphor screens for 12 h (Canberra-Packard, Ontario, Canada). The screens were then read by a Phosphor Imager scanner (Cyclone® Plus; PerkinElmer, USA).

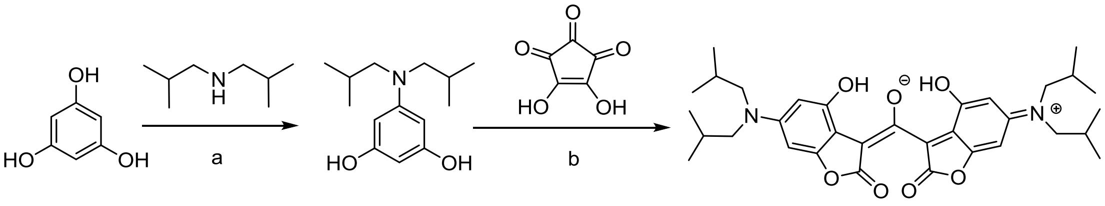


1 2 (A1094)

**Scheme S1.** Synthetic route of A1094. Regents and conditions. (a) Phloroglucinol and diisobutylamine (1:1, molar ratio) was condensed under azeotropic reflux in argon atmosphere for 6 h, brown-gray oily liquid solid, 92% yield; (b) The compound 1 and croconic acid (2:1, molar ratio) were azeotropic refluxed in DMSO for 2 h, black-purple solid, 7.8% yield.

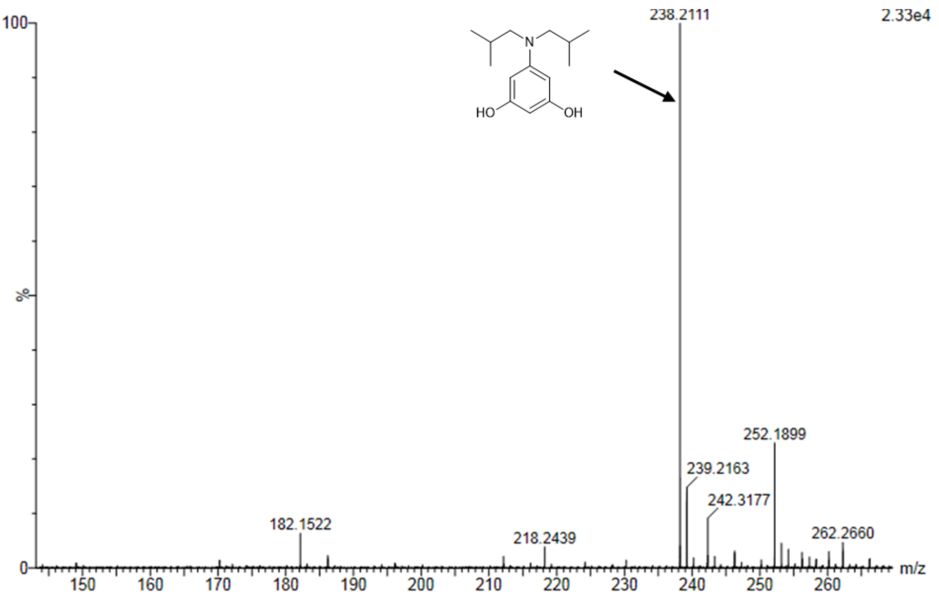


**Figure S1.** Mass spectrum of compound **1**. HRMS ($ESI^+$), calculated for $C_{14}H_{24}NO_2^+$, m/z 238.17, found 238.11.

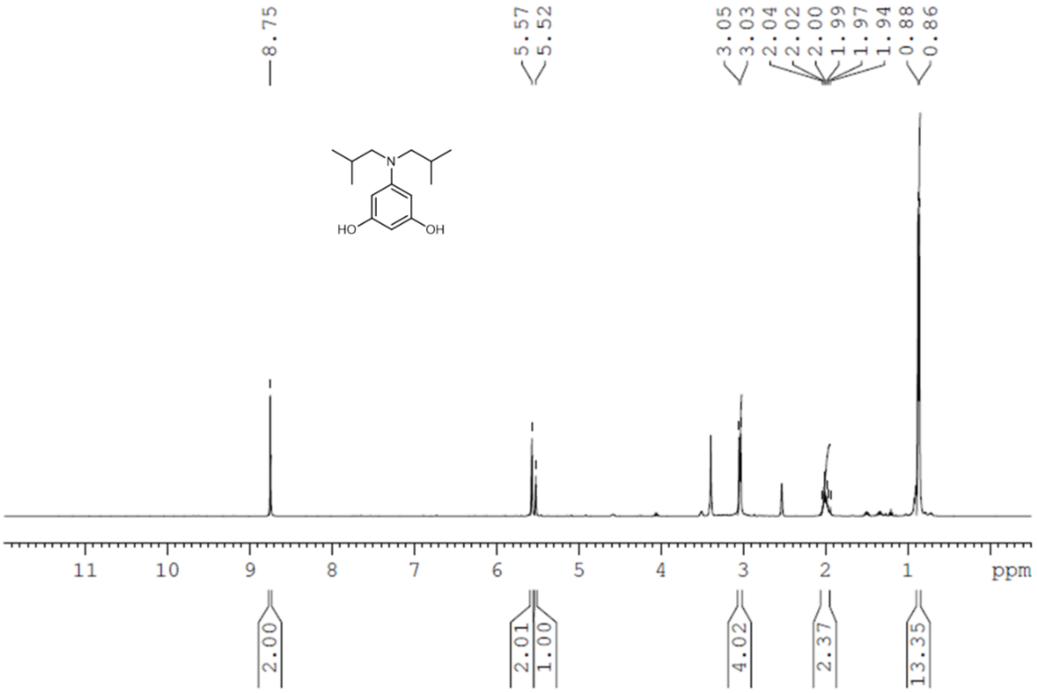


**Figure S2.** $^{1}$H NMR (DMSO-$d_6$, 400 MHz) spectrum of compound **1**.

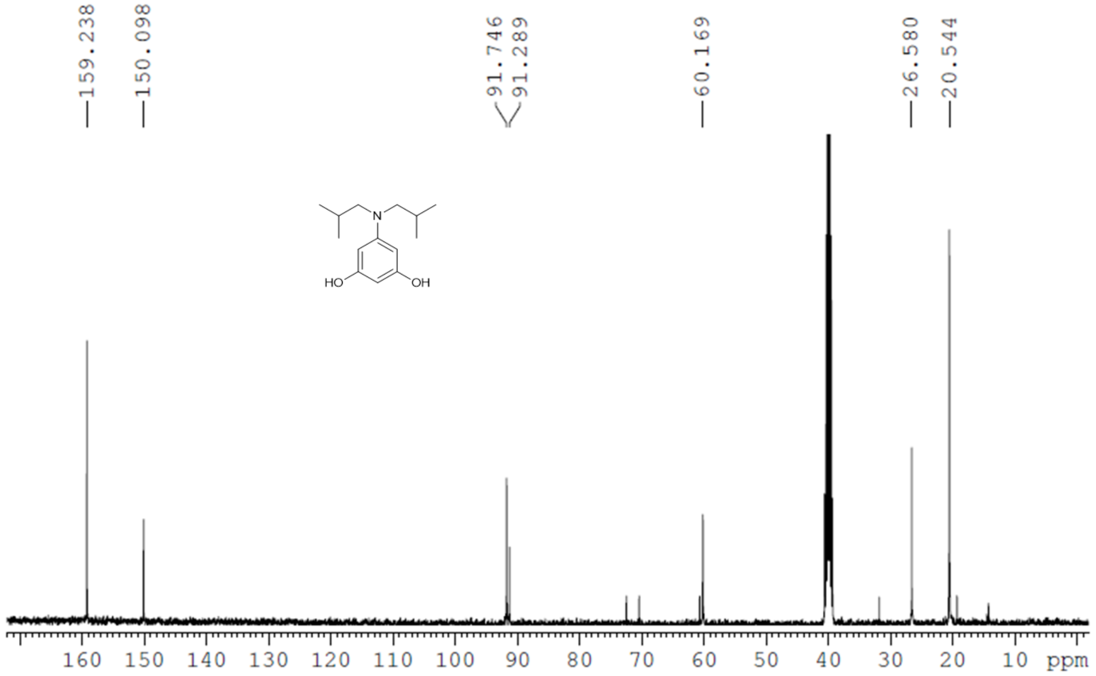


**Figure S3.** $^{13}C$ NMR (DMSO-$d_6$, 100 MHz) spectrum of compound **1**.

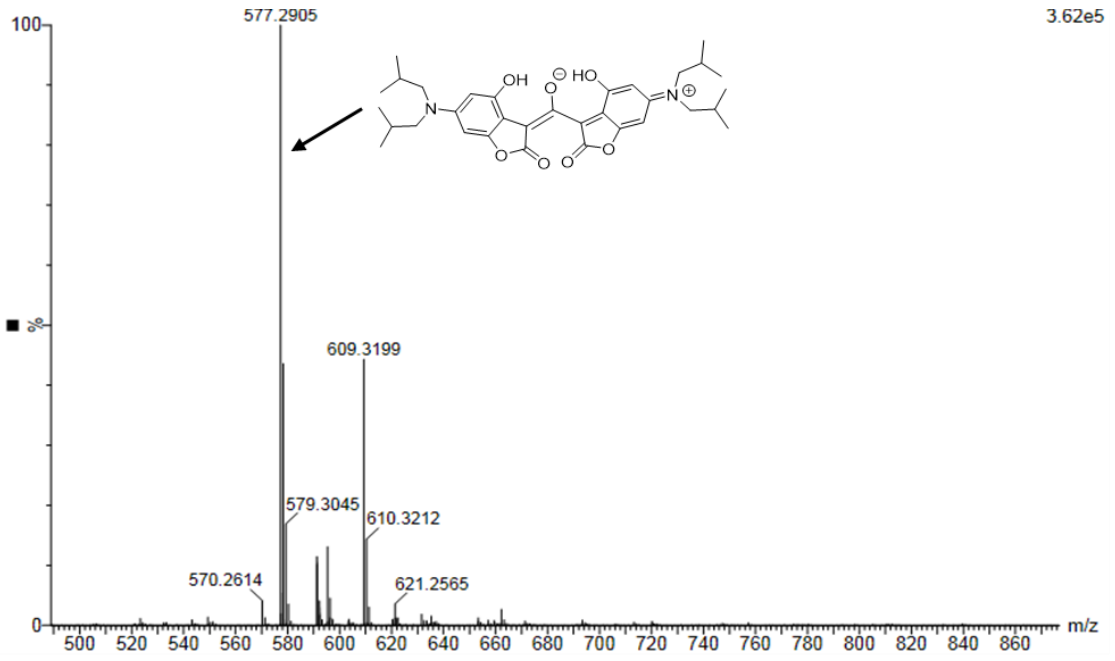


**Figure S4.** Mass spectrum of compound **2** (A1094). HRMS (ESI$^-$), calculated for $C_{33}H_{41}N_2O_7^-$, m/z 577.30, found 577.34.

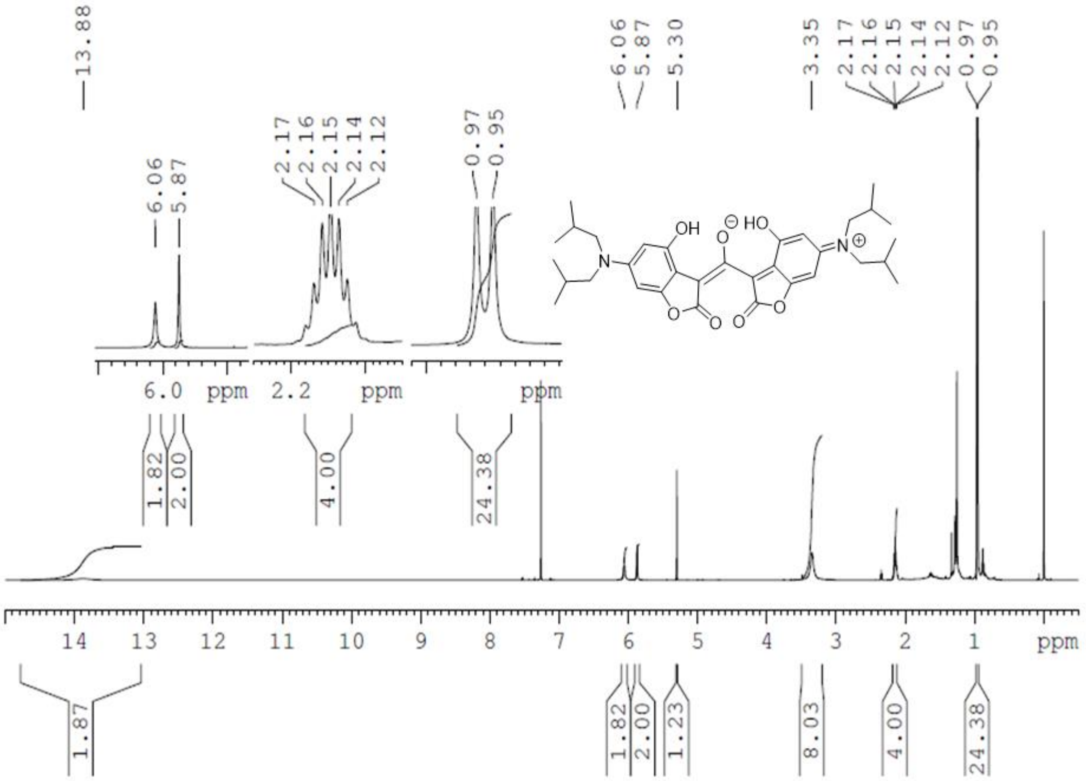


**Figure S5.** $^{1}$H NMR ($CDCl_3$, 600 MHz) spectrum of compound **2** (A1094).

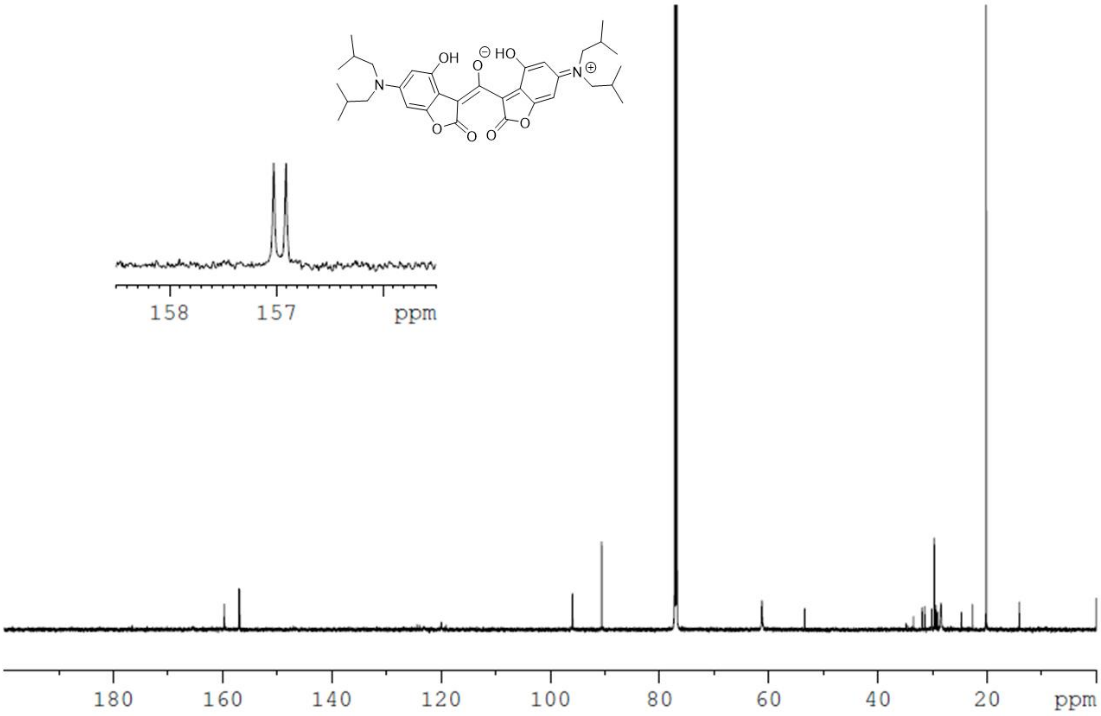


**Figure S6.** $^{13}$C NMR ($CDCl_3$, 150 MHz) spectrum of compound **2** (A1094).

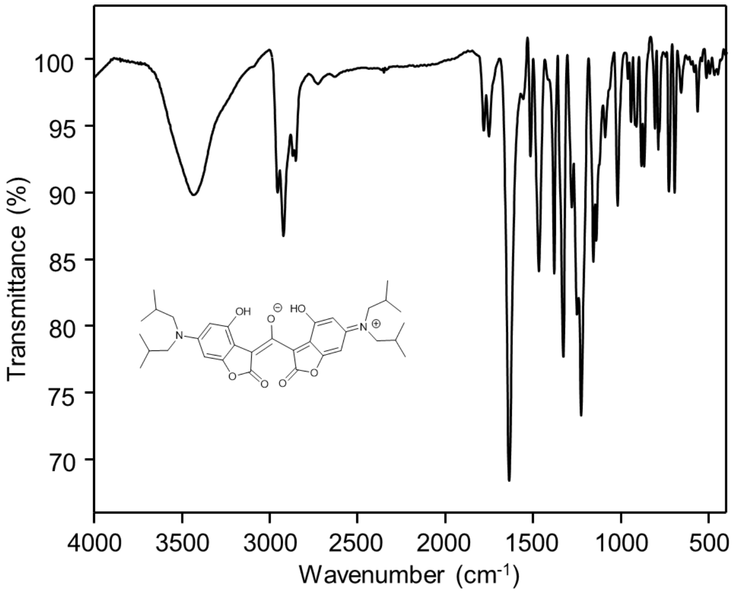


**Figure S7.** FTIR spectroscopy of compound **2** (A1094).

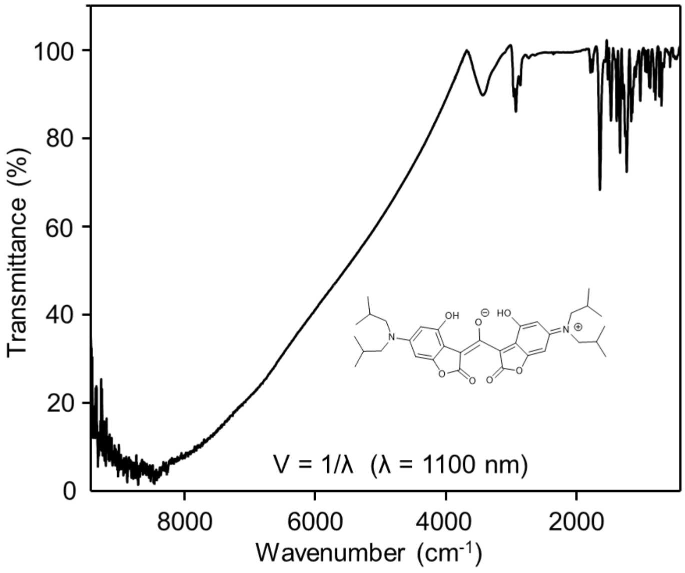


**Figure S8.** FTIR spectroscopy of compound **2** (A1094) in scope of 500 to 9500 $cm^{-1}$. The maximum transmission peak was in good agreement with the absorption at the molecular level and its absorption in NIR-II was described from the point of infrared spectroscopy.

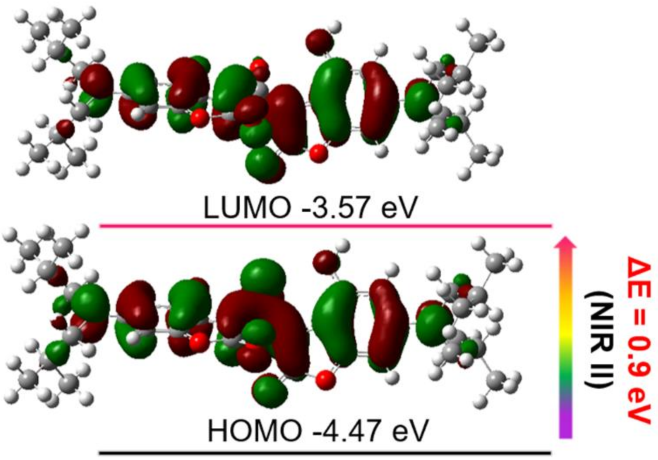


**Figure S9.** The density functional theory (DFT) calculation for the HOMO and LUMO of A1094 using B3LYP functional and 6-311G(d,p) basis set with the SMD model (dichloromethane solvent). This narrow gap confirmed its absorption in the NIR-II region.

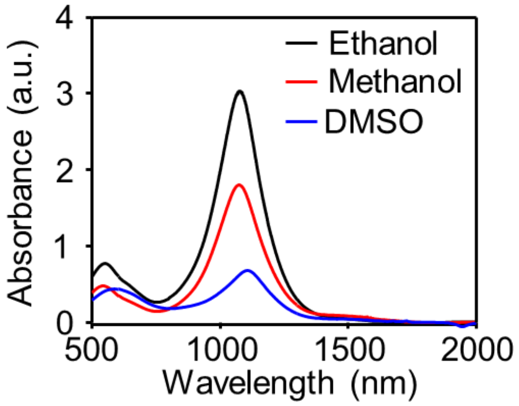


**Figure S10.** Absorption spectra of A1094 in different solutions (ethanol, methanol, DMSO) at the same concentration.

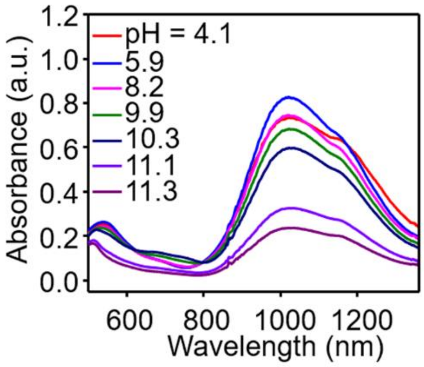


**Figure S11.** Absorption spectra of A1094 in different pHs at the same concentration.

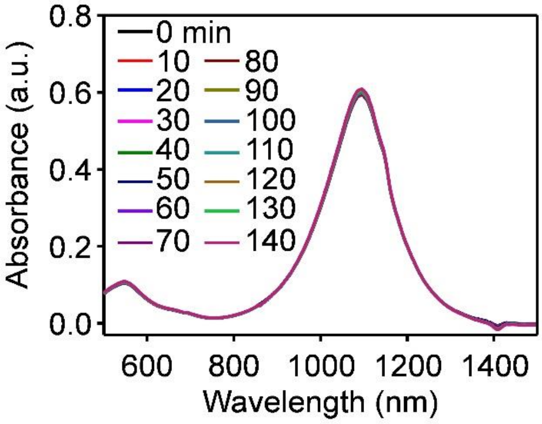


**Figure S12.** Light stability of A1094. Absorption spectra of A1094 monomers in dichloromethane. It was tested every ten minutes for 140 min, which displayed excellent light stability after 14 times light irradiation.

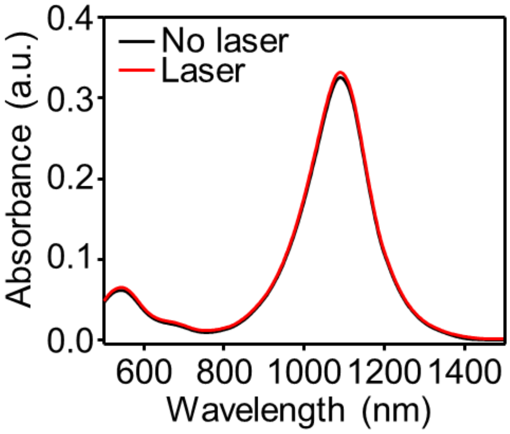


**Figure S13. Photothermal stability of A1094.** Absorption spectra of A1094 monomers in dichloromethane before and after being treated by 980 nm laser (0.5 W cm$^{-2}$, 10 min).

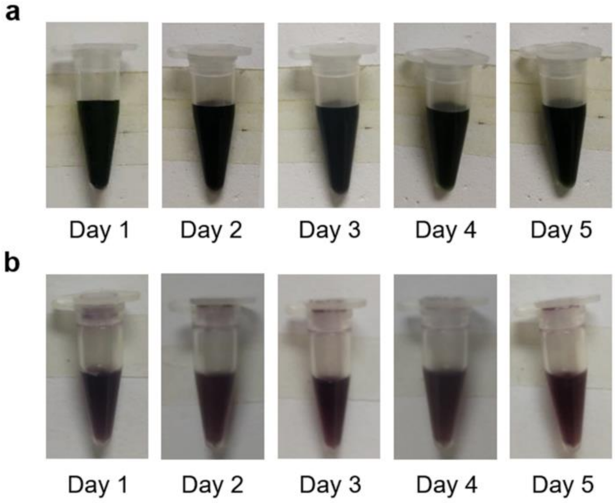


**Figure S14.** The stability of (a) A1094 in DMSO and (b) A1094@RGD-HBc in PBS at room temperature after 5 days' storage.

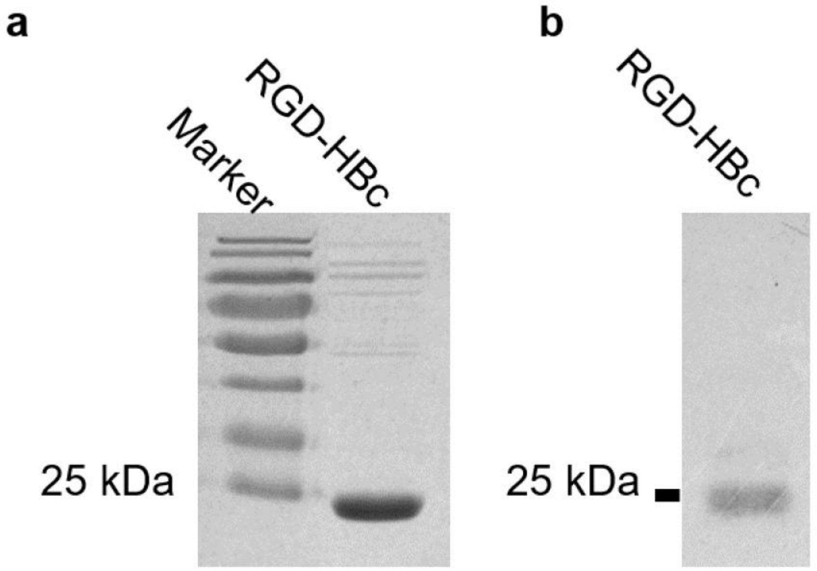


**Figure S15.** Expression of RGD-HBc-NS5A protein. (a) SDS-PAGE and (b) Western blotting of RGD-HBc-NS5A.

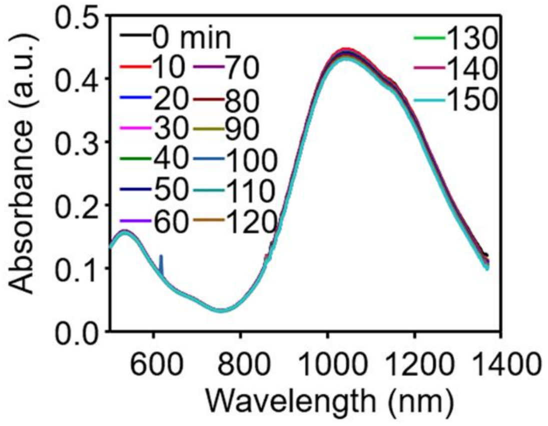


**Figure S16.** Absorption spectra of A1094@RGD-HBc during 150 min, which showed excellent light stability after 14 times light irradiation.

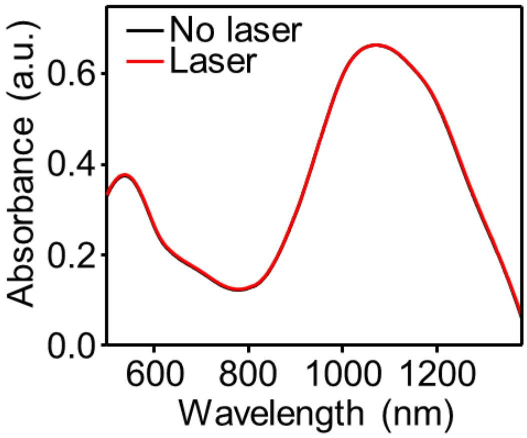


**Figure S17. Photothermal stability A1094@RGD-HBc.** Absorption spectra of A1094@RGD-HBc in PBS before and after being treated by 980 nm laser (0.5 W $cm^{-2}$, 10 min).

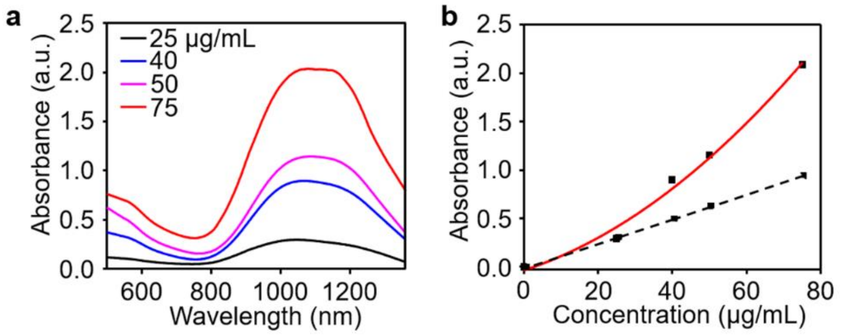


**Figure S18. (a)** Near infrared absorption of A1094@RGD-HBc with different concentrations (25, 40, 50, 75 μg/mL) of A1094. **(b)** The concentration absorption curve (red line) of A1094@RGD-HBc (the black line is the tangents of red line).

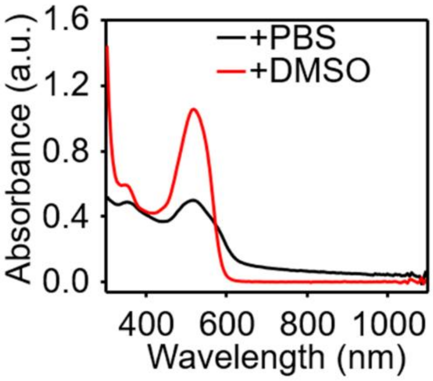


**Figure S19.** Absorption spectra of Oil Red O@HBc before and after DMSO destroying the protein.

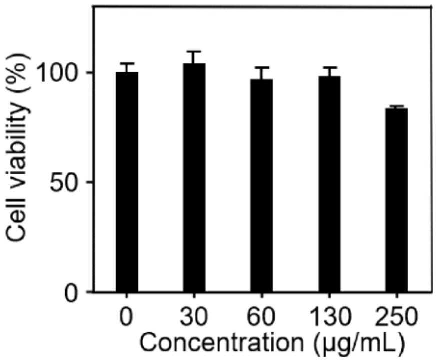


**Figure S20.** Cell viability of U87MG cells after incubation with A1094@RGD-HBc at different concentrations.

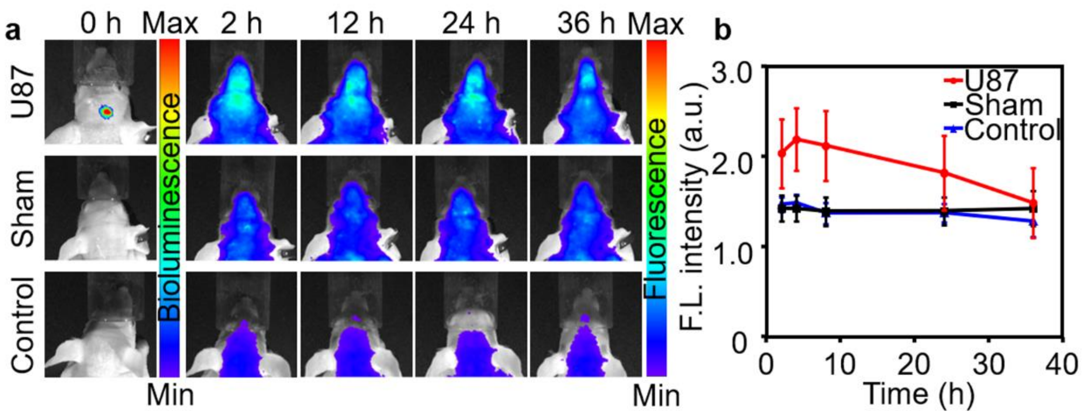


**Figure S21.** (a) Fluorescence images of the brain of the U87MG tumor-bearing, normal, and sham groups at different time points after injection of Cy5.5-A1094@RGD-HBc under 675 nm excitation. (b) Corresponding fluorescence intensities of (a).

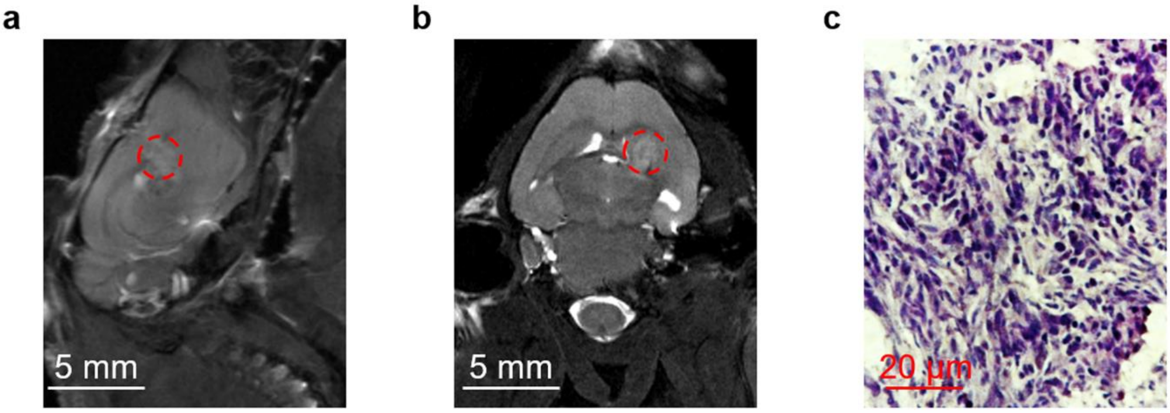


**Figure S22.** Diagnosis of brain cancer. (a) Sagittal and (b) coronal views of contrast-enhanced T2-weight MRI of an orthotopic tumor-bearing mouse. (c) H&E stain of the brain tumor section.

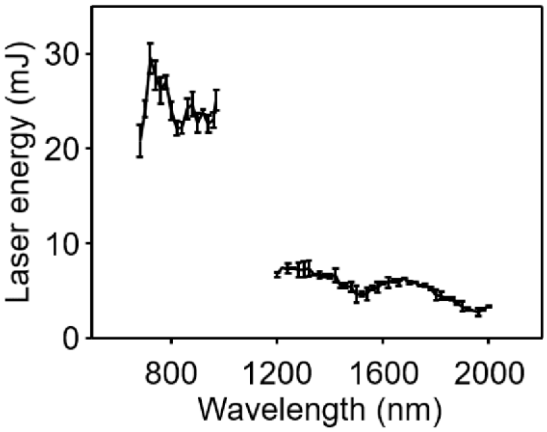


**Figure S23. The energy curves of photoacoustic at different wavelengths.** The energy values of laser below 1000 nm were approximately 5 times stronger than that above 1000 nm.

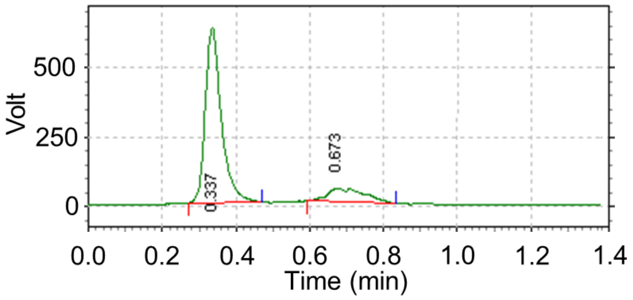


| Retention time | Compound | Area percentage | Height percentage |
|---|---|---|---|
| 0.337 | A1094@RGD-HBc | 84.722 | 92.752 |
| 0.673 | $^{131}$I | 15.278 | 7.248 |

**Figure S24.** The labeling rate of $^{131}$I-A1094@RGD-HBc nanoparticles. The left peak represented A1094@RGD-HBc compounds and the right peak was $^{131}$I. The labeling rate of $^{131}$I-A1094@RGD-HBc nanoparticles was 84.72%.

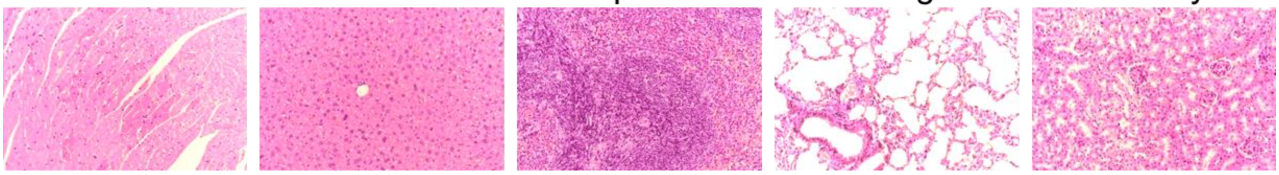


**Figure S25.** H&E stain showing major organs after 7 days of A1094@RGD-HBc administration (magnification: 200×). The results of H&E staining showed that the material was safe to the major organs of mice.

**Reference**

[1] L. Shen, J. Zhou, Y. Wang, N. Kang, X. Ke, S. Bi, L. Ren, *Small* **2015**, *11*, 1190.

**Author Contributions**

Y. L., Z. L. and L. N. conceived and designed the experiments. H. L. and W. S. prepared the materials. Y. L., H. L. and H. Y. performed the imaging processing. Y. L., L. R. and J. Z. processed experimental data. Y. L., H. L., Z. L. and L. N. wrote the manuscript.
